# Prognostic criteria in patients with gastrointestinal stromal tumors: a single center experience retrospective analysis

**DOI:** 10.1186/1477-7819-10-43

**Published:** 2012-02-20

**Authors:** Naoki Tanimine, Kazuaki Tanabe, Takahisa Suzuki, Noriaki Tokumoto, Hideki Ohdan

**Affiliations:** 1 Department of Surgery, Division of Frontier Medical Science, Graduate School of Biomedical Sciences, Hiroshima University, 1-2-3 Kasumi Minami-ku Hiroshima 734-8551, Japan

**Keywords:** gastrointestinal stromal tumor (GIST), prognostic criteria, recurrence, nomogram, adjuvant therapy

## Abstract

**Background:**

Gastrointestinal stromal tumors (GISTs) are morphologically and clinically heterogeneous tumors, and their biological behavior is difficult to predict, ranging from clinically benign to malignant. The aim of our study was to reanalyze the value of the commonly used prognostic criteria and recently reported nomogram in predicting disease recurrence in patients with primary resectable GISTs.

**Methods:**

The clinicopathological features of 60 patients with GISTs who underwent surgical resection between 1998 and 2010 at Hiroshima University Hospital were retrospectively reviewed. Tumors were classified according to the National Institutes of Health and Armed Forces Institute of Pathology criteria, and nomogram predictions were performed. The relationship between patient and tumor characteristics was tested by univariate analysis using the log-rank test. Furthermore, we assessed nomogram performance with the concordance index and calibration.

**Results:**

The median patient follow-up was 4.1 years, with 6 of 60 patients experiencing recurrence. Recurrence was observed only in the high-risk group. The recurrence-free survival (RFS) was 93.0 and 89.9% after 2 and 5 years, respectively. The concordance indices of the nomogram prediction were 0.96 and 0.65 for all patients and the high-risk subgroup, respectively. Calibration of the nomogram-predicted RFS tended to overestimate the recurrence risk relative to the actual RFS.

**Conclusions:**

Although the commonly used criteria provide an excellent estimation of tumor behavior, they are limited by prognostic heterogeneity. The predictive nomogram is a beneficial scoring system but not a direct RFS predictor. We need more consideration for small GISTs, particularly those less than 3 cm in diameter, and small GISTs should be analyzed as a subset with potentiality different biological behavior.

## Background

Gastrointestinal stromal tumor (GIST) is the most common mesenchymal neoplasm of the intestinal tract. The tumor typically occurs in the stomach or small intestine, infrequently in the colon, rectum, and esophagus, and rarely outside the gastrointestinal tract. The gold standard therapy for localized primary GIST is surgical resection [1,2]. Unfortunately, the results of surgery alone have been inadequate, with up to 50% of patients developing tumor recurrence within 5 years and eventually dying from the disease [3-5].

In 2000, imatinib mesylate (Novartis Pharmaceuticals, Basel, Switzerland) was found to be effective against metastatic GIST in the initial patient tested [6], and its efficacy was then confirmed in a phase II [7,8] and in phase III trials [9,10]. In 2009, the American College of Surgeons Oncology Group (ACOSOG) reported the results of study Z9001, a randomized control trial assessing the efficacy of adjuvant imatinib for patients with primary GISTs larger than 3 cm [11]. More recently, at the American Society of Clinical Oncology (ASCO) 47th Annual Meeting, the results of the SSG XVIII-AIO study were presented. This phase III trial revealed that 3 years of treatment with imatinib after surgery in patients with high-risk GIST according to the National Institutes of Health (NIH) criteria [12], including patients who had tumor rupture before or during surgery, improved overall and recurrence-free survival (RFS) compared to the finding after 1 year of treatment.

GISTs are morphologically and clinically heterogeneous tumors, and their biological behavior is difficult to predict, ranging from clinically benign to malignant. The NIH criteria are based on the evaluation of the size and mitotic rate of the tumors as the most reliable prognostic factors, and their use is common. Another set of commonly used criteria that considers a third prognostic factor--tumor location--was proposed by the Armed Forces Institute of Pathology (AFIP) [13,14]. In addition, Gold *et al. *reported that their prognostic nomogram provided a better prediction of the likelihood of recurrence for individual patients in Western datasets than the commonly used staging criteria that stratify patients into a few broad groups [15].

The aim of our study was to reanalyze the value of the prognostic criteria regarding their relationship to disease recurrence in patients with primary resectable GISTs in our prospectively collected tumor registry as a Japanese dataset.

## Methods

**Figure 1:**
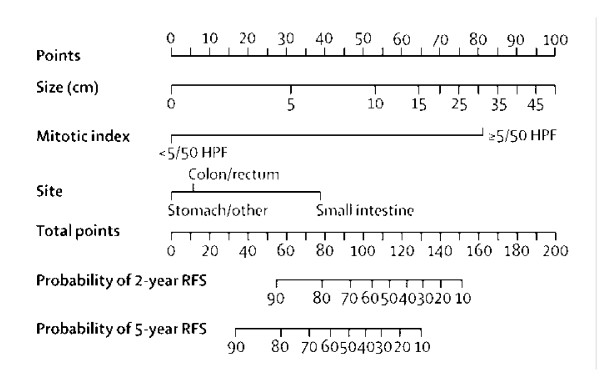
**Prognostic nomogram adapted from Gold *et al***. [**15**]. Points are assigned for size, mitotic index, and site of origin by drawing a line upward from the corresponding values to the "Points" line. The sum of these 3 points, plotted on the "Total points" line, corresponds to predictions of 2- and 5-year recurrence-free survival. HPF: high-power fields.

From 1998 to 2010, 60 patients presented to our institution with primary GIST without metastasis. Patient, tumor, and treatment data were collected prospectively. Complete gross resection of the tumor was performed in all patients. The technique of resection was at the discretion of the individual surgeon. An expert pathologist confirmed the diagnosis of GIST and calculated the mitotic index. The diagnosis of GIST was confirmed by immunohistochemical staining for CD117. The mitotic index was determined by counting the number of mitotic figures per 50 high-power fields (HPFs) and categorized as less than 5 or 5 or more mitoses. Size measurements were performed by the institutional pathologists, either before or after formalin fixation, and tumors were categorized as 5 cm or less or more than 5 cm in diameter. Tumors were classified according to the NIH and AFIP criteria, which are 2 commonly used sets of criteria (Table 1). Simultaneously, nomogram predictions were performed for the tumors [15]. The nomogram assigned points based on tumor size in a continuous but non-linear fashion. Points for tumor site were assigned on the basis of whether the tumor arose in the stomach, small intestine, colon/rectum, or an extraintestinal location, and points for mitotic index were assigned on the basis of whether the primary tumor had less than 5 or 5 or more mitoses per HPF (Figure 1). No patient was treated with a tyrosine kinase inhibitor before developing recurrence. As a follow up study, chest and abdominal computed tomography (CT) scans were performed at least every 6 months after surgery on patients with greater than intermediate- or moderate-risk, and at least every year on patients with very low- or low-risk. However, CT scans were repeated earlier whenever clinically indicated depending on the discretion of the investigator. Endoscopy was performed annually. Patients did not undergo any further selection. Follow-up information was obtained during regular outpatient visits or by phone with the patient and/or the referring physician. During follow-up, we analyzed the incidence of disease recurrence. All deaths from other causes were recorded. RFS was defined as the time from patient registration to the development of tumor recurrence.

**Table 1:** Commonly used criteria for assessing risk of GIST

NIH criteria^14^	
Very low	< 2 cm and < 5 mitotic index
Low	2-5 cm and < 5 mitotic index
Intermediate	5-10 cm and < 5 mitotic index
	> 5 cm and 6-10 mitotic index or
High	> 5 cm and > 5 mittic index or
	> 10 cm and any mitotic index or
	Any size and > 10 mitotic index
**AFIP criteria^16, 17^**	
Unknown *	Expect from following criteria
Very low	≦ 5 cm and ≦ 5 mitotic index
Low	Gastric: > 5 cm and ≦ 10 cm and ≦ 5 mitotic index
	Others: > 2 cm and ≦ 5 cm, and ≦ 5 mitotic index
Moderate	Gastric: > 10 cm and ≦ 5 mitotic index or
	> 2 cm and ≦ 5 cm, and > 5 mitotic index
	Others: > 5 cm and ≦ 10 cm, and ≦ 5 mitotic index
High	Gastric: > 5 cm and > 5 mitotic index
	Others: > 10 cm or > 5 mitotic index

* Category with small numbers of cases insufficient for prediction of malignant potential

NIH: the National Institutes of Health

AFIP: the Armed Forces Institute of Pathology

Definition for risk categories in the National Institutes of Health and Armed Forces Institute of Pathology criteria.

We estimated RFS probabilities with the Kaplan-Meier method. The relationships of patient and tumor characteristics to outcome were investigated by univariate analysis using the log-rank test. Multivariate analysis could not be performed because of the small number of recurrence events. A p-value of < 0.05 was considered statistically significant. SPSS statistical software (v.18; Chicago, IL, USA) was used for univariate analysis.

We assessed nomogram performance in 2 ways. First, the discriminatory capability of the nomogram was determined using the concordance index (C index) [16]. The interpretation of the C index is similar to that of the area under the receiver operating characteristic curve. The C index provides the probability that the nomogram will predict a poorer outcome for the patient who recurred first out of a randomly selected pair of patients. The C index was assessed with respect to 3 groups: all patients, a subgroup excluding very low- and low-risk patients, and a subgroup limited to patients whose tumors were classified as high-risk by either NIH or AFIP criteria. Second, calibration was evaluated by comparing the Kaplan-Meier-observed RFS for 4 quartiles of patients stratified by nomogram score 2 years after surgical treatment. Calibration was limited to the 2-year RFS because the median follow-up period was insufficient to estimate the 5-year RFS.

## Results

The detailed clinicopathological features of the patients with primary GISTs are shown in Table 2. The median age of the patients was 63 years (range, 18-83 years), and 31 patients (51.7%) were men. Tumor locations included the stomach, small intestine, and rectum in 48 (80%), 11 (18.3%), and 1 (1.7%) patient, respectively. The median tumor size was 3.8 cm (range, 1.6-20 cm). The median duration of follow-up for patients in this series was 4.1 years (range 0.1-12.8 years), with 6 of 60 patients experiencing recurrence. Five of six patients had disease recurrence in the liver, and another had local intrapelvic recurrence. All of these recurrences were detected by follow-up CT scan, with 4 of the recurrence events occurring less than 1 year after surgery. Three patients were lost to follow-up before 2 years. All 3 patients lost to follow-up had very low- or low-risk tumors. According to the NIH criteria, 34 (56.6%), 13 (21.7%), and 13 (21.7%) tumors were classified as very low- or low-, intermediate-, and high-risk, respectively. According to the AFIP criteria, 3 (5.0%), 35 (58.3%), 13 (21.7%), and 9 (15%) tumors were classified as unknown, very low or low, moderate, and high risk, respectively. In the univariate analysis, size and the mitotic index predicted RFS (p = 0.002). When correlating recurrence with tumor location, a trend toward statistical significance became evident (p = 0.051).

**Table 2:** Characteristics of 60 patients with primary resectable GISTs

Clinicopathological feature	n (total = 60)	Recurrence events (n = 6)	univariate analysis P value
**Follow up period (years)**			
median (range)	2.8 (0.1-11.8)		
**Sex**			
Female	29	2	
Male	31	4	0.465
**Age (years)**			
median (range)	63 (18-83)		
≦ 63	31	5	
> 63	29	1	0.211
**Tumor location(%)**			
Stomach	48 (80)	3	
Others	12 (20)	3	**0.51**
**Tumor size (cm)**			
median (range)	3.8 (1.6-20)		
≦ 5	43	1	
> 5	17	5	***0.002***
Mitotic index*			
< 5	39	0	
≧ 5	21	6	***0.002***
**Resection margin**			
R0	51	4	
R1	9	2	0.156
**NIH criteria (%)**			
Very low & Low	34 (56.6)	0	
Intermediate	13 (21.7)	0	
High	13 (21.7)	6	
**AFIP criteria (%)**			
Unknown	3 (5.0)	0	
Very low & Low	35 (58.3)	0	
Moderate	13 (21.7)	0	
High	9 (15)	6	

*Mitotic index = number of mitoses per 50 high-power fields.

NIH the National Institutes of Health

AFIP the Armed Forces Institute of Pathology

Detailed clinicopathological features of the patients with primary GISTs and the results of univariate analysis.

**Figure 2:**
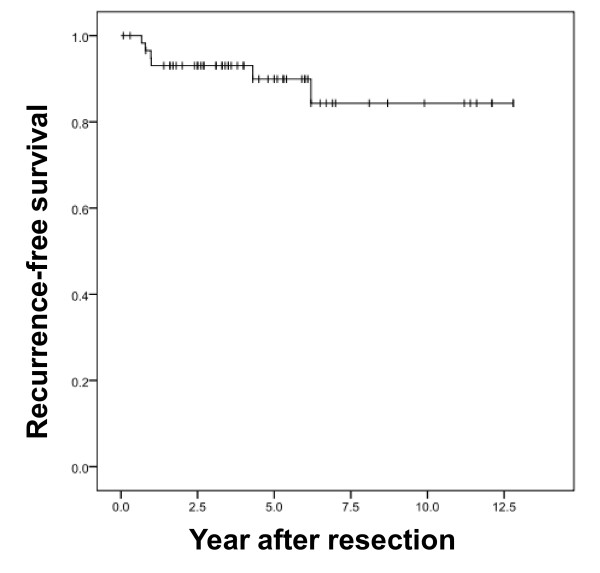
**Recurrence-free survival of total patients**. Kaplan-Meier estimates of the recurrence-free survival of patients with primary GIST after complete surgical resection.

**Figure 3:**
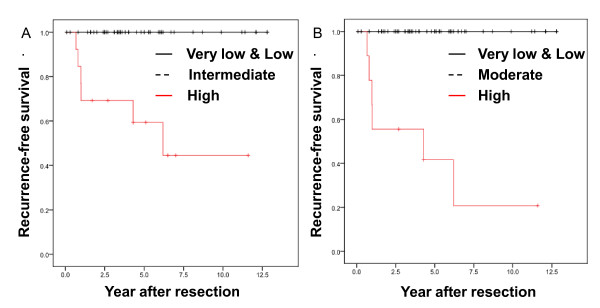
**Recurrence-free survival classified using commonly used criteria**. **A**. Kaplan-Meier estimates of recurrence-free survival of primary resectable GIST patients classified according to NIH criteria. **B**. Kaplan-Meier estimates of recurrence-free survival of primary resectable GIST patients classified according to AFIP criteria.

RFS was 93.0% (SE 0.034%), and 89.9% (SE 0.045%) after 2 and 5 years, respectively (Figure 2). In our series, the 2-year and 5-year RFS was better than that reported previously. RFS-classified risk groups according to the NIH and AFIP criteria are shown in Figure 3. Recurrence events were observed only in the groups classified as high risk by either set of criteria.

**Figure 4:**
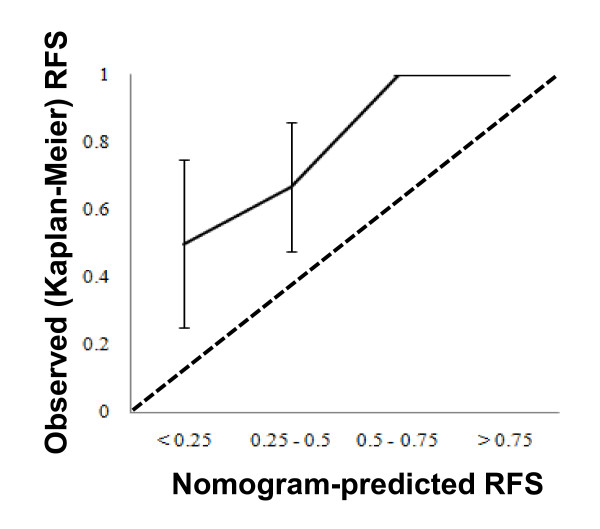
**Calibration of nomogram-predicted recurrence-free survival (RFS)**. Observed RFS is shown compared with nomogram-predicted RFS at 2 years.

Next, we estimated the discriminatory capability of the nomogram by using the C index. The C index of the nomogram prediction for all patients was 0.96, which was adequately acceptable. The C indices of the nomogram predictions excluding the low-risk subgroup and limited to only the high-risk subgroup were 0.91 and 0.65, respectively. Therefore, in 65% of the cases, the nomogram correctly predicted the order of outcome between 2 randomly selected patients who were classified as high-risk according to either the NIH or AFIP criteria. A calibration test was performed to estimate the accuracy of the RFS predicted by the nomogram. Calibration of the nomogram-predicted RFS tended to overestimate recurrence compared with the Kaplan-Meier-observed RFS (Figure 4).

## Discussion

In our series, 2-year and 5-year RFS was better than that previously reported (93.0% and 89.9%, respectively). There were a large proportion of very low- and low-risk patients. The reasons for the large proportion of low-risk GISTs may be the excellent screening system and the early indication for surgery. Simply, this may mean that early diagnosis and resection improve the overall survival. Meanwhile, our dataset had an obviously high proportion of smaller tumors than Western datasets. Because of their high mitotic indices, some "small" GISTs less than 3 cm in diameter were classified as intermediate- or high-risk tumors. The proportion of "small" GISTs may account for the better prognosis compared to those observed in Western datasets.

Currently, accurate prognostication of GISTs is essential, not only in guiding the clinician regarding the frequency and intensity of postoperative surveillance but also, to enable better selection of tumors for potential adjuvant treatment. In current clinical practice, relatively large numbers of clinicians appear to recommend adjuvant imatinib therapy for patients with high-risk tumors according to the NIH criteria. In the ACOSOG Z9001 trial, one of the few studies of adjuvant imatinib therapy, patients were only stratified according to tumor size, which may not be the only prognostic factor in recent studies, making it difficult to adequately select patients for whom the adjuvant treatment could be clearly beneficial. In 2010, at the Gastrointestinal Cancers Symposium (ASCO-GI), Blackstein *et al. *reported a stratified analysis of the Z9001 trial using the AFIP criteria. [17] The 2-year RFS of the low-risk patients was 98% and thus, there was no benefit of adjuvant therapy. The recurrence rate of the high-risk patients selected with 3 factors--tumor size, mitotic index, and tumor location--was the highest and the high-risk patients gained the greatest effect from imatinib adjuvant therapy. The importance of these 3 factors was also suggested in our present study. Simultaneously, recurrence events were observed only in the group classified as high risk by both the NIH and AFIP criteria and many of these events occurred during the first postoperative period. This result indicates that commonly used criteria provide an excellent estimation of tumor behavior. However, regarding adjuvant therapy, the high-risk patients classified by the commonly used criteria do not always include patients who can benefit fully from the use of adjuvant therapy. Huang *et al. *clearly revealed the limitations of these criteria [4]. The high-risk category has been criticized as being too heterogeneous. According to the results of the SSG XVIII-AIO study, if a long duration of adjuvant therapy is recommended to all high-risk patients, the adverse effects of imatinib adjuvant therapy are not negligible. Considering the cytostatic effect of imatinib, it is speculated that the best indications for long adjuvant therapy are patients who are expected to develop recurrence in the early period after surgery. We experienced 3 "small" GISTs less than 3 cm in diameter that were classified as high-risk GISTs according to the NIH or AFIP criteria. The patients with these tumors had no recurrences at 1.7, 4.3, and 6.5 years after surgery, respectively. In a practical sense, it is difficult to decide whether adjuvant therapy is necessary for these patients. Naturally, the potential for recurrence and metastasis is lower for smaller tumors. In particular, the Z9001 trial did not reveal the benefit of adjuvant imatinib for small GISTs less than 3 cm, and this minor subset has not been analyzed adequately in Western countries. It is expected that adjuvant therapy for GISTs will be more individualized. Concerning adjuvant therapy, we need more consideration for small GISTs, and small GISTs should be analyzed as a subset with potentially different biological behavior.

Nomograms can estimate tumor size in a continuous but nonlinear fashion and calculate the risk of recurrence at a point in time for any individual patient. No other staging system has been assessed for its ability to assign a quantitative risk of recurrence for individual patients. It might be challenging to justify the use of adjuvant imatinib by nomogram prediction alone because the nomogram prediction overestimated the recurrence risk compared with the actual RFS in our series. However, the discriminatory capability of the nomogram for the subgroup of high-risk patients is worthy of attention (C index = 0.65).

When interpreting the results of the current analysis, it is important to consider the limitation of our dataset. The small sample size of a single center experience is not sufficient to validate and decide the cut-off value. However, we can suggest the nomogram as a beneficial scoring system in practical situations, but not as a direct RFS predictor.

In addition, the prognostic criteria could be improved with the incorporation of additional variables, for example, mutation status. Gold *et al. *failed to observe an improvement in the accuracy of the nomogram prediction when mutation status was included [15]. However, conflicting results exist about whether KIT and platelet-derived growth factor receptor alpha (PDGFRA) mutation status affect outcome among patients with resected localized primary GISTs [18-26]. Approximately 85% of GISTs contain an activating mutation in the KIT proto-oncogene, whereas 3-5% of patients have a PDGFRA mutation [12,27,28]. Moreover, the effect of imatinib varies depending on the domains of KIT and PDGFRA affected by the mutations. Therefore, many uncertainties remain regarding techniques to assess prognostic factors, including whether tumor size should be measured before or after fixation and whether the most mitotically active tumor areas should be assessed, as well as regarding the dosage and duration of imatinib adjuvant therapy. Given these uncertainties, longer follow-ups and results from additional trials are needed.

## Conclusions

Although the commonly used criteria provide an excellent estimation of tumor behavior, they are limited by the prognostic heterogeneity of their high-risk tumor categories. The predictive nomogram is a beneficial scoring system but not a direct RFS predictor. We need more consideration for small GISTs, particularly those less than 3 cm in diameter, and small GISTs should be analyzed as a subset with potentially different biological behavior.

## List of abbreviations

GIST: gastrointestinal stromal tumors; RFS: recurrence-free survival; ACOSOG: American College of Surgeons Oncology Group; NIH: National Institutes of Health; HPFs: high-power fields; AFIP: Armed Forces Institute of Pathology; C index: concordance index; PDGFRA: platelet-derived growth factor receptor alpha.

## Competing interests

The authors declare that they have no competing interests.

## Authors' contributions

NT collected data, performed analysis, and drafted, revised and finalized the manuscript. KT conceived this study and participated in its design and coordination. TS, NT, and HO revised and approved the contents of the manuscript. All authors read and approved the final manuscript.

## Authors' information

Department of Surgery, Division of Frontier Medical Science, Graduate School of Biomedical Sciences, Hiroshima University
